# Supplementary material for: Npbwr1 signaling mediates fast antidepressant action
Source: Mol Psychiatry. 2024 Oct 21;30(5):1828–35. doi: 10.1038/s41380-024-02790-4 (PMC12015170; doi:10.1038/s41380-024-02790-4)
Supplement: Supplementary file 1 — Supplementary material [file 41380_2024_2790_MOESM1_ESM.docx]

**Supplementary information**

**
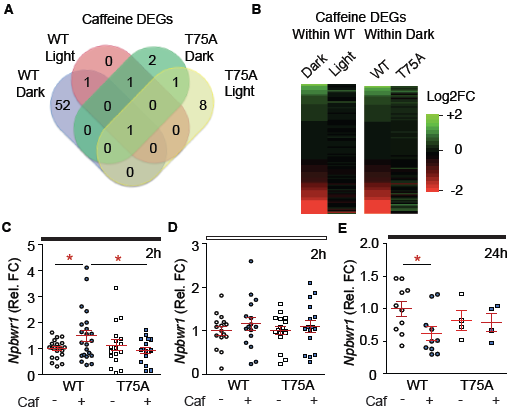
**

**Supplementary Fig. 1: RNA-sequencing identifies *Npbwr1* as a target of caffeine and the DARPP-32 pathway. A**, **B**) RNA-sequencing on the NAc of male wildtype (WT) and DARPP-32-T75A mutant mice (T75A) at the end of the active (ZT18) and in the middle of the inactive phases (ZT0) of the light cycle, 2 h after caffeine injection. Caffeine was thus injected at ZT0-2h, ZT6-2h, ZT12-2h and ZT18-2h. These time points were chosen based on previously measured, diurnal effects of caffeine on mood and gene expression, which were prevented by T75A-DARPP-32 ^12^. **A**) Venn diagrams of significant (Padj < 0.05, log2FC 0.5 <> -0.5) gene expression changes between various conditions. These data confirm that the biggest effect of caffeine occurs in the active phase of WT mice. **B**) Heat maps comparing transcriptional changes (log(FC) across light phases and genotypes All logFC-changes were included. **C**-**E**) Validation in a different cohort by qPCR. **C**) Dark phase. 2 h after injection, caf increases *Npbwr1* in WT but not T75A mutants. n = 16-22; interaction drug & genotype: F(1,73) = 4.54, P < 0.05; *post hoc* test: drug effect within WT: *P < 0.05; genotype effect within caf: *P < 0.05. **D**) No effects on *Npbwr1* 2 h after caffeine during the light phase. n = 16,16,17,16; genotype effect: F(1,53) = 0.06, P = 0.79; drug effect: F(1,53) = 1.10, P = 0.29; interaction: F(1,53) = 0.06, P = 0.80. **E**) Dark phase. *Npbwr1* is reduced in WT 24 h after caffeine injection. n = 10,10,4,4; genotype effect: F(1,24) < 0.01, P = 0.98; drug effect: F(1,24) = 2.02, P = 0.17; interaction: F(1,24) = 1.53, P = 0.23; *post hoc* test: drug effect within WT: *P < 0.05. **C**-**E**) Independent data points are plotted and means **±** s.e.m. are shown. DEGs: differentially expressed genes; Non-significant comparisons are not listed unless specified.

**
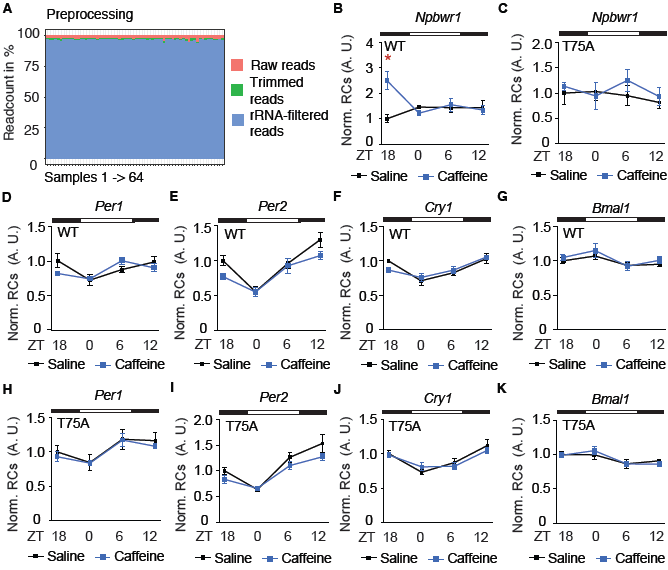
**

**Supplementary Fig. 2: Quality control markers and read count distribution of RNA-sequencing after acute caffeine treatment. A**) Preprocessing information on RNA quality. All samples reached at least 90% alignment. **B, C**) Distribution of *Npbwr1* read-counts (normalized for WT ZT18) over time. As depicted in **Fig. S1**, the biggest change in *Npbwr1* occurs at the end of the dark phase in caffeine-treated WT mice. **B**-**K**) n = 4 for all groups. **B**) WT; drug effect: F(1,24) = 1.38, P < 0.05; *post hoc* test: effect of caffeine within ZT18: *P < 0.05. Sal: ZT18 vs. ZT0: P < 0.001; ZT18 vs. ZT6, 12: P < 0.01. **C**) T75A; drug effect: F(1,24) = 0.76, P = 0.39; time effect: F(3,24) = 0.57, P = 0.63; interaction: F(3,24) = 0.36, P = 0.78. **D**-**K**) Normalized read counts (RCs) for circadian genes. Circadian alterations are in agreement with the literature. Caffeine but not T75A-mutation may slightly flatten the amplitude of circadian oscillations. **D**-**G**) WT. **D**) *Per1*; time effect: F(3,24) = 5.05, P < 0.01; *post hoc* test: Sal: ZT18 vs. ZT0: P < 0.01; ZT0 vs. ZT12: P < 0.05; Caf: ZT0 vs. ZT6: P < 0.05. **E**) *Per2*; drug effect: F(1,24) = 5.35, P < 0.05; time effect: F(3,24) = 23.82, P < 0.0001; *post hoc* test: Sal: ZT18 vs. ZT0, ZT0 vs. ZT12, ZT6 vs. ZT12: P < 0.001; ZT18 vs. ZT12: P < 0.05; ZT0 vs. ZT6: P < 0.01; Caf: ZT18 vs. 12: P < 0.05; ZT0 vs. ZT6: P < 0.01; ZT0 vs. ZT12: P < 0.001. **F**) *Cry*; time effect: F(3,24) = 13.19, P < 0.0001; *post hoc* test: Sal: ZT0 vs. ZT6: P < 0.01; ZT0 vs. ZT6, ZT6 vs. ZT12: P < 0.05; Caf: ZT0 vs. ZT12: P < 0.05; ZT0 vs. ZT12: P < 0.001; ZT6 vs. ZT12: P < 0.01. **G**) *Bmal*; time effect: F(3,24) = 3.48, P < 0.05; *post hoc* test: Caf: ZT0 vs. ZT6: P < 0.05. **H**-**K**) T75A. **H**) *Per1*; time effect: F(3,24) = 5.14, P < 0.01; *post hoc* test: Sal, Caf: ZT0 vs. ZT6: P < 0.05. **I**) *Per2*; drug effect: F(1,24) = 4.9, P < 0.05; time effect: F(3,24) = 26.49, P < 0.0001; *post hoc* test: Sal: ZT18 vs. ZT0: P < 0.05; ZT18 vs. ZT12, ZT0 vs. ZT6, ZT0 vs. ZT12: P < 0.001; Caf: ZT18 vs. ZT12, ZT0 vs. ZT6: P < 0.01; ZT0 vs. ZT12: P < 0.0001. **J**) *Cry*; time effect: F(3,24) = 13.76, P < 0.0001; *post hoc* test: Sal: ZT18 vs. ZT0, ZT6 vs. ZT12: P < 0.01; ZT0 vs. ZT12: P < 0.001; Caf: ZT0 vs. ZT12: P < 0.01; ZT6 vs. ZT12: P < 0.05. **K**) *Bmal*; drug effect: F(1,24) < 0.01, P = 0.99; time effect: F(3,24) = 5.73, P < 0.01; interaction: F(3,24) = 0.45, P = 0.72; *post hoc* test: Caf: ZT0 vs. ZT6, ZT0 vs. ZT12: P < 0.05. **B**-**K**) Means **±** s.e.m. are shown. ZT: Zeitgeber time. Non-significant are comparisons not listed unless specified.

**
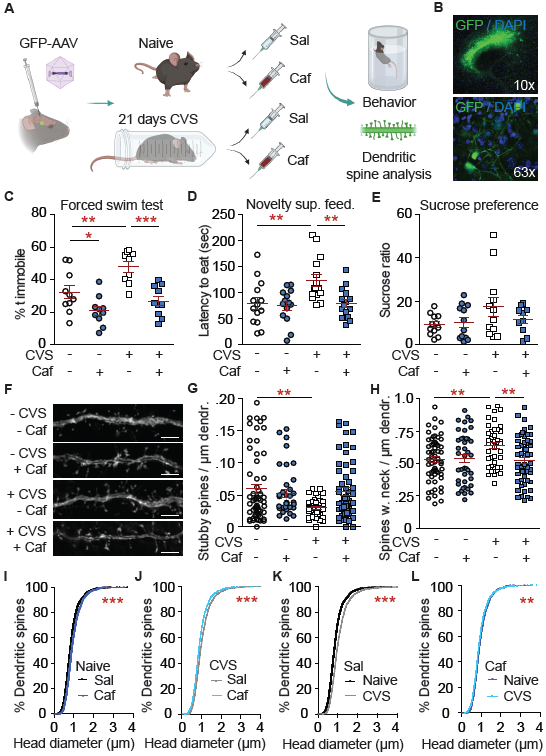
**

**Supplementary Fig. 3: Acute caffeine injection reverses CVS-induced changes in depressive-like behaviors and dendritic spine changes in females. A**) Experimental overview. Given the mood-elevating effects of caffeine we previously detected ^12^, its’ effects were now assessed in a chronic stress mouse model. A subgroup of mice was stereotaxically injected with a GFP-expressing AAV for dendritic spine analysis. **B**) Overview image of GFP-labelled neurons. **C**-**E**) Stress-induced behavioral changes are reversed 24 h after caffeine injection. **C**) Caf reduces immobility time in the forced swim test in stressed and unstressed mice. n = 10,10,9,10; stress effect: F(1,35) = 10.38, P < 0.01; drug effect: F(1,35) = 23.36, P < 0.0001; *post hoc* test: caf effect within naïve: *P < 0.05, within CVS: ***P < 0.001; stress effect within sal: **P < 0.01. **D**) Caf rescues stress effects on latency to eat in the Novelty-suppressed feeding test. n = 14,14,15,15; stress effect: F(1,54) = 6.21, P < 0.05; drug effect: F(1,54) = 6.17, P < 0.05; interaction: F(1,54) = 4.28, P < 0.05; *post hoc* test: drug effect within CVS: **P < 0.01; stress effect within sal: **P < 0.01. **E**) No significant changes were measured in the Sucrose preference test within this cohort. n = 12,12,12,10; stress effect: F(1,42) = 2.91, P = 0.10; drug effect: F(1,42) = 0.72, P = 0.40; interaction: F(1,42) = 1.61, P = 0.21. **F**-**N**) CVS-induced changes in NAc dendritic spines are reversed by caffeine. **F**) Representative dendrites. Scale bar 10 μm. **G**) Stubby spines. n = 59,38,42,58 dendrites from 6,4,4,6 mice; stress effect: F(1,192) = 4.85, P < 0.05; interaction: F(1,192) = 6.73, P < 0.05; *post hoc* test: n.s. **H**) Neck-containing spines. n = 60,39,42,60 dendrites from 6,4,5,6 mice; drug effect: F(1,196) = 4.86, P < 0.05; interaction: F(1,196) = 5.77, P < 0.05; *post hoc* test: drug effect within CVS: **P < 0.01; stress effect within sal: **P < 0.01. **I**-**L**) Cumulative head diameter (HD) is increased by CVS and reversed by caf. **I**) Caf increases HD within naïve mice: χ^2^ = 65.36, df = 1, ***P < 0.0001. **J**) Caf reduces HD within CVS group: χ^2^ = 51.74, df = 1, ***P < 0.0001. **K**) CVS increases HD within sal group: χ^2^ = 141.90, df = 1, ***P < 0.0001. **L**) CVS slightly decreases HD within caf group: χ^2^ = 7.04, df = 1, **P < 0.01. **C**-**E**, **G**, **H**) Independent data points are plotted and means **±** s.e.m. are shown. Non-significant comparisons are not listed.

**
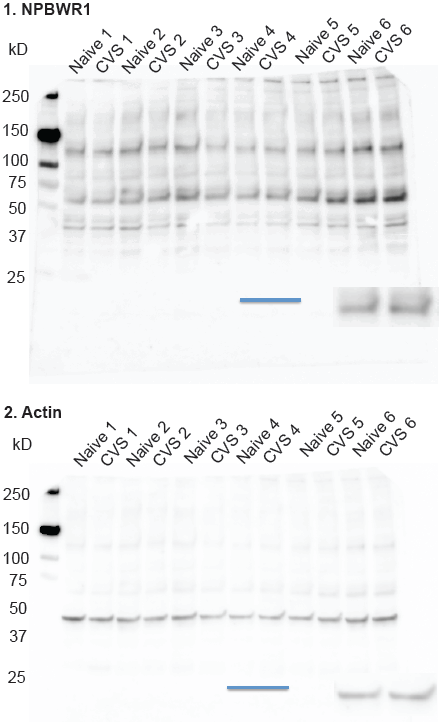
**

**Supplementary Fig. 4: Full-length western blots of NPBWR1 and Actin control.**

**
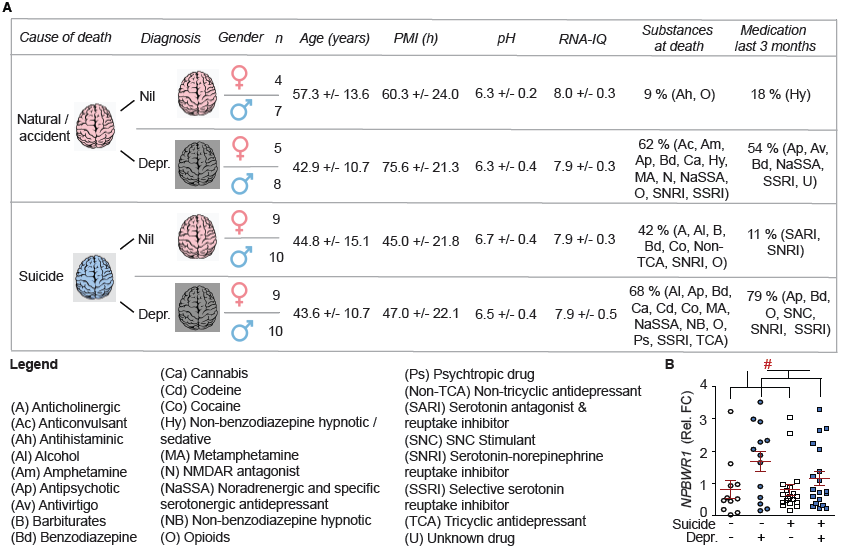
**

**Supplementary Fig. 5: Demographics of postmortem samples and separation by cause of death. A**) Patient information. **B**) Analysis split by cause of death and diagnosis. n = 11,13,19,19; 2-way ANOVA: effect of ilness: F(1,65) = 5.45, ^#^P < 0.05; effect of cause of death: F(1,65) = 0.38, P = 0.36; interaction: F(1,65) = 0.66, P = 0.42; *post hoc* test: all comparisons P > 0.05. Independent data points are plotted and means **±** s.e.m. are shown. PMI: postmortem interval; RNA-IQ: RNA IQ Score. Sketches were made with biorender.com. Nil: controls. Depr.: Depression.


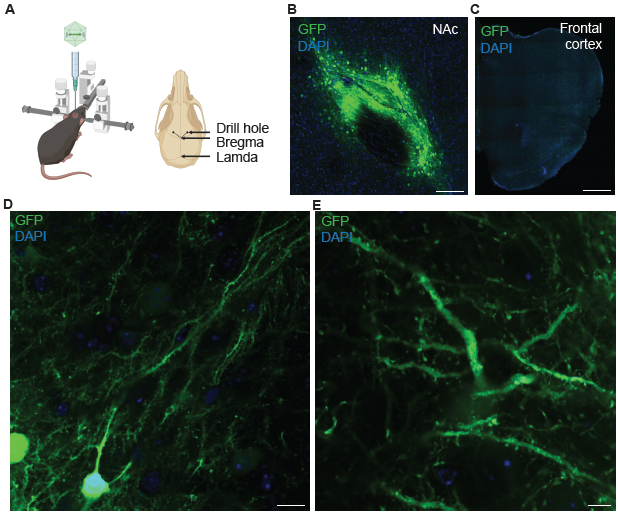


**Supplementary Fig. 6: Viral infections. A**) Overview of stereotaxic injection. **B**) Injection was selectively targeting the NAc. Scale bar: 100 μm. **C**) No signal was detected in the frontal cortex. Scale bar: 400 μm. **D**, **E**) Higher magnification reveals that the AAVs predominantly label medium spiny neurons, as evident by the arborous, spiny dendrites. **D**) Scale bar: 25 μm. **E**) Scale bar: 10 μm. Sketch was generated with biorender.com.

**
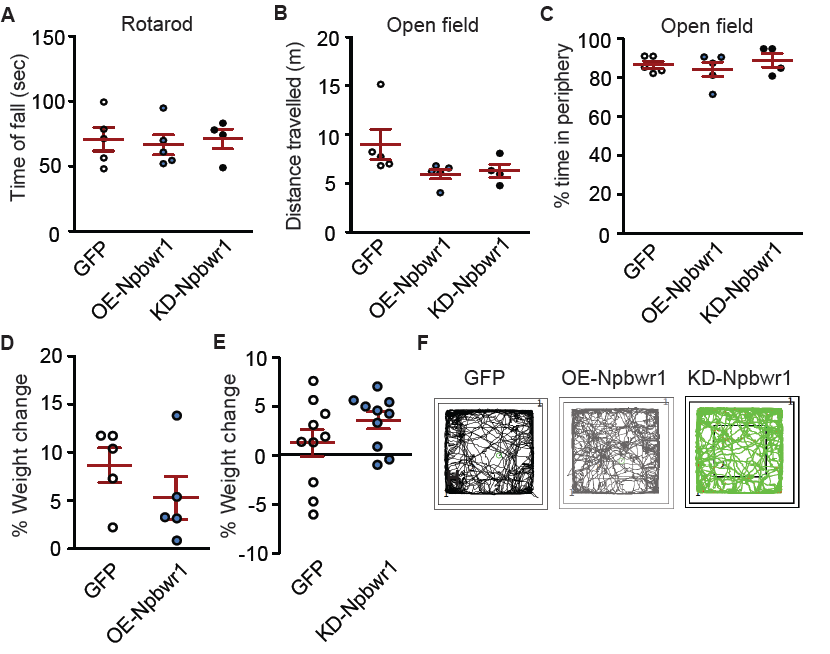
**

**Supplementary Fig. 7: No effects on weight, locomotor activity, or anxiety after altered *Npbwr1* levels. A**-**F**) Mice were stereotaxically injected with AAVs overexpressing (OE) or knocking down (KD) *Npbwr1* and analyzed at least 4 weeks later. **A**) No affect of *Npbwr1* on time to fall from a rotarod: n = 5,5,4; F(2,13) = 0.1, P = 0.91. **B**) Total distance traveled in the open field is not affected by altered *Npbwr1*: n = 5,5,4; F(2,13) = 2.51, P = 0.13. **C**) Time spent in the periphery of an open field is not affected by *Npbwr1*: n = 5,5,4; F(2,13) = 0.54, P = 0.60. **D**) No effect of OE-*Npbwr1* on weight gain after surgery. n = 5; t_8_ = 1.17, P = 0.28. **E**) KD-*Npbwr1* did not significantly affect weight change after surgery. n = 10; t_18_ = 1.39, P = 0.18. **F**) Examples of infected mice from all 3 groups moving in the open field arena. **A**-**E**) Independent data points are plotted and means **±** s.e.m. are shown. Non-significant comparisons are not listed unless specified. Sketches were made with biorender.com.


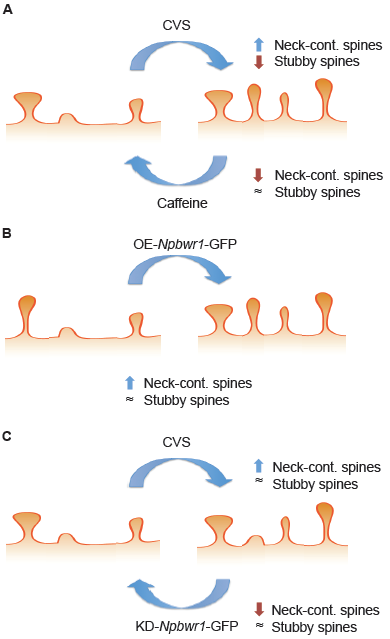


**Supplementary Fig. 8: Overview over observed changes in dendritic spines. A**) Experiment from **Fig. S3** (CVS and 24 h of caffeine treatment). **B**) Experiment from **Fig. 2** (OE-*Npbwr1*). **C**) Experiment from **Fig. 3** (KD-*Npbwr1*). **A**-**C**): CVS increased the density of neck-containing spines (“thin”and “mushroom” spines), which was mimicked by OE-*Npbwr1*. This increase was reversed by caffeine (**A**) and prevented by KD-*Npbwr1*. **A**-**C**) In our hands, stubby spine density was mostly unaffected, or reduced by CVS. Stubby spines are functionally mute, small protrusions of the dendrites and only represent a small % of spines.

**
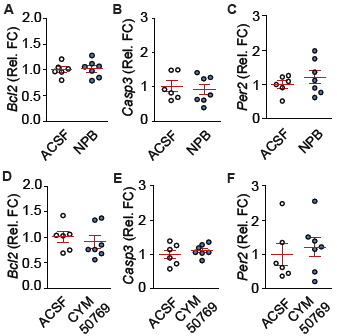
**

**Supplementary Fig. 9: Acute microinjection of *Npbwr1*-ligands is selective and non-toxic. A**-**C**) 1nmolar NPB **D**-**F**) 1 μmolar CYM50769 was microinjected into the NAc. Tissue was collected 24 h later and analyzed by qPCR. n = 6,7. Average +/- s.e.m. shown. **A**) No effect of NPB on *Bcl2*. P = 0.882. **B**) NPB does not alter *Casp3*. P = 0.66. **C**) *Per2* is not affected by NPB. P = 0.38. **D**) No effect on *Bcl2*. P = 0.58. **E**) CYM50769 does not alter *Casp3*. P = 0.42. **F**) *Per2* is not affected by CYM50769. P = 0.63.

**
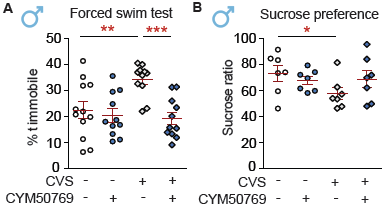
**

**Supplementary Fig. 10: CYM50769 rapidly reverses stress-induced changes in male mice.**

**A**) Rescued reduction in immobility time in the forced swim test. n = 12,11,11,11; effect of CVS: F(1,41) = 10.64, P < 0.01; effect of CYM50769: F(1,41) = 4.13, P < 0.05; interaction: F(1,41) = 6.26, P < 0.05; *post hoc* test: CVS effect within control group: **P < 0.01; CYM50769 effect within CVS-treated groups : ***P < 0.001. **B**) CVS effect on sucrose preference is blocked in mice that received CYM50769. n = 7 for all groups; interaction between CVS and CYM50769: F(1,42) = 6.09, P < 0.05; *post hoc* test: CVS effect within control group: *P < 0.05.


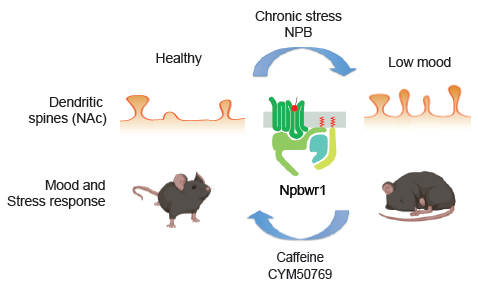


**Supplementary Fig. 11:** Overview of proposed mechanisms. Chronic stress and depression increase *Npbwr1* levels, while NPB stimulates *Npbwr1* activity, leading to low mood and an increased density of neck-containing spines in the NAc. Chronic stress effects can be prevented by reducing *Npbwr1* levels, using slower viral approaches or acute caffeine administration, or by decreasing *Npbwr1* activity via the fast-acting antagonist CYM50769. Sketches were made with biorender.com.

**Supplementary Methods**

**Primers**

| **Organism** | **Gene** | **Sequence** |
| --- | --- | --- |
| Mouse | *Actin* Fvd | 5'-TGTTACCAACTGGGACGACA-3' |
| Mouse | *Actin* Rev | 5'-GGGGTGTTGAAGGTCTCAAA-3' |
| Mouse | *Bcl2* Fvd | 5'-TACCGTCGTGACTTCGCAGAG-3' |
| Mouse | *Bcl2* Rev | 5'-GGCAGGCTGAGCAGGGTCTT-3' |
| Mouse | *Bdnf* Fvd | 5'-AAAATGCTCACACTCCAC-3' |
| Mouse | *Bdnf* Rev | 5'-GAACAAATGCTGGTCTTT-3' |
| Mouse | *Bmal1* Fvd | 5'-CAACCTTCCCGCAGCTAACA-3' |
| Mouse | *Bmal1* Rev | 5'-TCCGCGATCATTCGACCTA-3' |
| Mouse | *Casp3* Fvd | 5'-AGGGGTCATTTATGGGACA-3' |
| Mouse | *Casp3* Rev | 5'-TACACGGGATCTGTTTCTTTG-3' |
| Mouse | *Cry1* Fvd | 5'-CCTCTGTCTGATGACCATGATGA-3' |
| Mouse | *Cry1* Rev | 5'-CCCAGGCCTTTCTTTCCAA-3' |
| Mouse | *Gapdh* Fvd | 5'-AACTTTGGCATTGTGGAAGG-3' |
| Mouse | *Gapdh* Rev | 5'-ACACATTGGGGGTAGGAACA-3' |
| Mouse | *Npbwr1* Fvd | 5'-GCTCAGTATGCCCCACCTTT-3' |
| Mouse | *Npbwr1* Rev | 5'-AGGCTATGAGGCAGGAAGGA-3' |
| Mouse | *Per1* Fvd | 5'-ACCAGCGTGTCATGATGACATAC-3' |
| Mouse | *Per1* Rev | 5'-GTGCACAGCACCCAGTTCCC-3' |
| Mouse | *Per2* Fvd | 5'-GAAAGCTGTCACCACCATAGAA-3' |
| Mouse | *Per2* Rev | 5'-AACTCGCACTTCCTTTTCAGG-3' |
| Human | *ACTIN* Fvd | 5'-GTTGCTATCCAGGCTGTGCT-3' |
| Human | *ACTIN* Rev | 5'-GAGGGCATACCCCTCGTAGA-3' |
| Human | *GAPDH* Fvd | 5'-TGGGCAGCCGTTAGGAAAG-3' |
| Human | *GAPDH* Rev | 5'-AGTTAAAAGCAGCCCTGGTGA-3' |
| Human | *NPBWR1* Fvd | 5'-AGGCTATGAGGCAGGAAGGA-3' |
| Human | *NPBWR1* Rev | 5'-AAGCCCAGGTAATCCACAGC-3' |

**Extended methods**

**AAVs & stereotaxic surgery.** For bilateral stereotaxic surgery into the NAc, mice were analgized with melosus 0.5 mg/ml (#120, CP-Pharma) and anesthetized using 1-1.5% isoflurane. Eyes were protected with Vitamycin cream (#CP3920, CP-Pharma). The skin on the skull was opened and small holes were drilled near the stereotaxic X/Z coordinates of the NAc (coordinates relative to Bregma: antero-posterior (Z): + 1.5, mediolateral (X): ±1.5, dorsoventral (Y): -4.4, angle: 10°). AAVs were injected at a rate of .1 μL/min per hemisphere for 5 min using 10 μL Gastight Hamilton syringes model 1801 RN. Mice were scored for general health and behavior at least 3 days post-surgery, receiving melosus daily.

**Cloning.** The Npbwr1 coding sequence was amplified by PCR from mouse brain cDNA and cloned in an AAV shuttle vector that contains GFP fused to a P2A sequence (Kim et al 2011, PMID 21602908) under the control of the CAG promoter (pAAV-CAG-GFP.P2A.Npbwr1-WPRE.SV40PolyA). To perform short hairpin mediated knockdown of Npwr1, a shRNA containing the Npwr1 specific sequence 5’- GGACGCCTTATCACCTGAGTA -3’ was introduced in an AAV shuttle vector that drives expression of the shRNA by the human U6 promoter and expression of the reporter GFP by a CAG promoter (pAAV-U6.Npwr1-CAG-GFP-WPRE3). A scrambled shRNA cassette of rat-Clathrin served as control.

**Stress induction by chronic variable stress.** To induce stress in mice, a chronic variable stress (CVS) protocol was employed over a period of 21 days. Each day, mice were subjected to a randomly selected stressor for one hour from a predetermined set, ensuring that no single stressor was used on consecutive days and that each stressor was applied an equal number of times throughout the study. All procedures were conducted during the light phase of the day-night cycle. The stressors employed in the CVS protocol included:

*Mild Electric Shocks*: Mice were placed in a chamber commonly used for fear conditioning experiments and subjected to 100 randomly administered mild electric shocks at an intensity of 0.45 mA for 1 h. The chamber accommodated up to five mice simultaneously.

*Tail Suspension*: Mice were secured for one hour by adhesive tape to a mast approximately 50 cm high, forcing them into an uncomfortable position.

*Tube Restraint*: Mice were placed in a perforated 50 ml Falcon tube or a similar tube within their home cage for one hour. This tube restricted their movement, simulating a confined environment while allowing them to maintain a normal sitting posture.

**Behavioral Tests.** Behavioral tests were conducted at the end of the dark phase of the day-night cycle. All experiments were performed blinded and under red light conditions to minimize disruption and stress to the animals.

The *Tail Suspension Test* was utilized to assess escape behavior from an unusual and uncomfortable situation, specifically a suspended vertical posture. During the Tail Suspension Test, mice were suspended by their tails for a duration of 6 min, during which they exhibited escape attempts characterized by negative geotaxis. The primary outcome measure was the duration of immobility, defined as the time during which the mice remained passive and refrained from climbing attempts.

The *Forced Swim Test* aimed to quantify escape behavior from an uncomfortable situation, serving as a marker for stress-related behavior, particularly reduced motivation. During the Forced Swim Test, mice were placed in a water bath for 6 min, during which they attempted to escape. The water bath consisted of a transparent cylinder made of plastic, with water maintained at a temperature of 19-22°C. Mice, like most mammals, are naturally capable of swimming, making this behavior a part of their normal repertoire. The test indirectly measured escape behavior by recording the duration of immobility, defined as the time during which the mice ceased active attempts to escape.

The objective of the *Splash Test* was to evaluate grooming behavior in mice, which is typically reduced in stress models. In this test, a few milliliters of liquid (10% sucrose) were sprayed onto the backs of the mice, and the percentage of time spent grooming within a 6-minute period, as well as the latency to initiate grooming, were recorded.

The *sucrose preference test* aimed to measure anhedonia. Forty-eight hours before the test, the mice were housed with two water bottles to acclimate them to two liquid sources. On the test day, the mice were single-caged and provided with one bottle containing plain water and another containing an 8% sucrose solution. The positions of the bottles were randomly assigned and equally distributed among all groups. After 24 h, the consumption of both solutions was measured, and the ratio of sucrose to water consumption was calculated.

The *novelty suppressed feeding test* assessed a combination of anhedonia and anxiety towards unfamiliar situations. Mice were placed on a restricted diet overnight (approximately 12 h), during which all food was removed from the cages. Following this period, each mouse received 1g of food, equivalent to approximately one-third of their normal consumption. To ensure consistent food distribution, mice were individually housed for the duration of the experiment. Subsequently, the latency for each mouse to initiate feeding on a food pellet in a novel cage was recorded, with a maximum observation time of 10 min. After completing the test, mice were allowed ad libitum access to food.

For the *open field test*, mice were recorded for 10 min in an arena with open space (approximately 50 cm x 50 cm) enclosed by walls. The distance traveled by each mouse was measured, and their frequency of staying in the center versus the periphery of the arena was assessed. This behavior evaluated the mice's tendency for risk-taking, as they often perceive the brightly illuminated center as more hazardous due to increased visibility to potential predators. The experiment was conducted under white light conditions, due to its ability to induce greater anxiety in mice compared to red light.

To measure locomotor skills, the mice were positioned on a rod within the *Rotarod* apparatus, initiating rotation at a predetermined speed. The start button was engaged only after ensuring the mice were securely settled on their rods. The duration until each mouse fell from the rotating rod was documented. Beneath the rod, approximately 50 cm above the ground, padding material was installed to provide a cushioned landing. Each mouse underwent the experiment three times, and the average time to fall was computed.
